# Advances in calcium signalling research for the diagnosis and treatment of depression

**DOI:** 10.1080/07853890.2026.2620329

**Published:** 2026-03-05

**Authors:** Lin Li, Mingli Yao, Jing Meng, Zhen Zhong, Nan Nan, Amir Hooman Kazemi, Jiale Guo, Chenyue Liu, Youming Jiang

**Affiliations:** a Beijing Key Laboratory of Psychoactive Substances Discovery and Control in Chinese Herbal Medicines, Institute of Chinese Materia Medica, China Academy of Chinese Medical Sciences, Beijing, China; b School of Traditional Chinese Medicine, Beijing University of Chinese Medicine, Beijing, China; c School of Life Sciences, Beijing University of Chinese Medicine, Beijing, China; d School of Persian Medicine, Tehran University of Medical Sciences, Tehran, Iran; e Guang’anmen Hospital, China Academy of Chinese Medical Sciences, Beijing, China; f The Third Affiliated Hospital of Beijing University of Chinese Medicine, Beijing, China

**Keywords:** Depression, calcium channels, diagnosis and treatment, pathophysiological mechanisms, therapeutic targets and strategies

## Abstract

**Background:**

The increasing global burden of depression underscores the need for novel therapeutic strategies beyond conventional antidepressants such as selective serotonin reuptake inhibitors and serotonin–norepinephrine reuptake inhibitors. Given the substantial role of calcium signaling in the pathogenesis of psychiatric disorders, this review examines its critical involvement in depression to guide future research and clinical advancements.

**Results:**

We describe the activation mechanisms of key calcium pathways, including voltage-gated channels, N-methyl-D-aspartate receptor–gated channels, endoplasmic reticulum–mediated release, and store-operated calcium entry, and summarize evidence of their dysregulation in clinical depression and animal models.Furthermore, we discuss the potential of calcium signaling as a diagnostic and prognostic biomarker, highlighting how emerging insights in this field may support the development of targeted antidepressant therapies.

**Conclusions:**

This review indicates that calcium signaling demonstrates potential as a diagnostic biomarker and a basis for targeted antidepressant therapies.

## GRAPHICAL ABSTRACT

**Figure:**
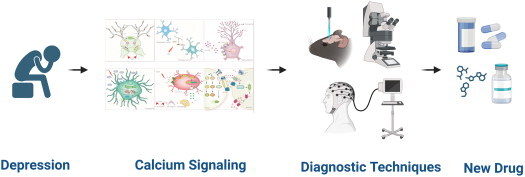

## 1. Introduction

Depression is a highly prevalent mental disorder worldwide, characterized primarily by persistent low mood [1]. Between 1990 and 2019, the global working population experienced a 44.79% increase in depression prevalence, accompanied by a 40.3% increase in incidence [2]. Disability-adjusted life years have increased by approximately 8.68 million, and the continual growth in annual prevalence places a significant burden on healthcare systems globally. Current antidepressant medications, including selective serotonin reuptake inhibitors (SSRIs) and serotonin–norepinephrine reuptake inhibitors (SNRIs), provide clinical benefit for many patients. However, these treatments often require up to 6 weeks to achieve therapeutic effectiveness, are frequently associated with adverse effects, and may necessitate combination therapy with multiple psychotropic medications [3,4]. Therefore, the identification of safer and more effective antidepressant strategies remains an urgent clinical priority.

The calcium signalling pathway is a fundamental regulator of neuronal function and plays a critical role in neurotransmitter release, synaptic plasticity, neuronal excitability, gene transcription, neuronal survival, and apoptosis. Its dysregulation is closely linked to the pathogenesis of several psychiatric disorders [5]. Calcium signalling involves a series of intracellular responses triggered by fluctuations in intracellular and extracellular calcium ion concentrations. Extracellular calcium enters neurons primarily through four pathways: voltage-gated calcium channels (VGCCs); receptor-gated channels such as N-methyl-D-aspartate (NMDA) and α-amino-3-hydroxy-5-methyl-4- isoxazolepropionic (AMPA) receptors (NMDARs/AMPARs); and store-operated calcium entry (SOCE). Intracellular calcium stores, including the endoplasmic reticulum (ER) and mitochondria, release calcium *via* inositol 1,4,5-trisphosphate (IP_3_) receptors (IP_3_Rs) and ryanodine receptors (RyRs). Calcium signalling is tightly regulated by buffering and clearance mechanisms consisting of calcium-binding proteins, pumps, and exchangers. VGCCs, IP_3_Rs, and RyRs control calcium flux, whereas calmodulin (CaM) activates downstream effectors such as calcineurin (CaN) and Ca^2+^/CaM-dependent protein kinase II (CaMKII). Homeostasis is maintained through the actions of the sarcoplasmic reticulum/ER Ca^2+^-ATPase (SERCA) pump, the plasma membrane calcium ATPase (PMCA), and the sodium–calcium exchanger. Together, these systems coordinate calcium release and clearance, with CaM, CaN, and CaMKII serving as key effectors within the calcium signalling pathway.

Dysregulation of neuronal calcium homeostasis is a key mechanism in the pathogenesis of depression, contributing to several downstream pathological processes, including synaptic loss, reduced neurogenesis, neuroinflammation, and hypothalamic–pituitary–adrenal (HPA) axis dysfunction [6,7]. Thus, exploring therapeutic strategies that restore calcium balance holds significant promise for improving depression outcomes. This review examines the role of calcium signalling in the pathogenesis and treatment of depression across four dimensions: activation pathways of calcium signalling, patterns of calcium signalling dysregulation in depressive states, pathophysiological mechanisms linking aberrant calcium signalling to depressive pathology, and the diagnostic and therapeutic potential of targeting calcium signalling. Through this review, we aim to provide fresh insights and novel therapeutic strategies for the development of antidepressants in both basic and clinical research on depression.

## 2. Activation mechanisms of calcium signalling pathways

### 2.1. VGCCs

VGCCs couple membrane depolarization to calcium influx, thereby initiating essential neuronal processes such as neurotransmitter release, excitation–contraction coupling, gene expression, and other downstream physiological responses. Based on their electrophysiological and pharmacological properties as well as differences in their α1 subunits, VGCCs are classified into five major channel types: Cav1.1–Cav1.4 (L-type Ca^2+^ currents), Cav2.1 (P/Q-type), Cav2.2 (N-type), Cav2.3 (R-type), and Cav3.1–Cav3.3 (T-type) [8,9]. Different VGCC subtypes operate in distinct neuronal compartments and mediate specialized physiological functions [10]. N-type and P/Q-type channels predominantly regulate neurotransmitter release at presynaptic terminals. Conversely, L-type VGCCs (L-VGCCs) are heteromeric complexes composed of pore-forming α1 subunits and auxiliary subunits, and they primarily mediate calcium entry in neuronal somata and dendrites. Among these, Cav1.2 and Cav1.3 are the major L-type Ca^2+^ channels expressed in the mammalian brain [11].

### 2.2. NMDA receptor–mediated channels

NMDA receptor–mediated calcium channels are ligand-gated ion channels composed of heterotetrameric complexes that include the essential GluN1 subunit together with GluN2 (GluN2A–D) or GluN3 subunits. Binding of glutamate, the primary excitatory neurotransmitter, along with the co-agonists glycine or D-serine, induces conformational changes that remove the Mg^2+^ block from the receptor pore. Upon subsequent membrane depolarization, the NMDA receptor channel opens, permitting the influx of calcium and sodium ions. This influx is crucial for neuronal activation and synaptic plasticity induction [12].

### 2.3. Mechanisms of calcium release from the ER

In addition to the influx of extracellular calcium mediated by VGCCs and NMDARs during action potentials and glutamatergic transmission, the ER serves as a major intracellular calcium store capable of releasing calcium to support neuronal activity. Two principal ER membrane channels—IP_3_Rs and RyRs—mediate this calcium release. Activation of G protein–coupled receptors (GPCRs) or receptor tyrosine kinases stimulates phospholipase C (PLC), which catalyzes the production of IP_3_. IP_3_ then binds to IP_3_Rs, triggering the release of calcium from the ER lumen into the cytosol [13]. RyRs play an equally essential role by amplifying calcium signaling through calcium-induced calcium release (CICR) [14,15].

### 2.4. SOCE

**Figure 1.**
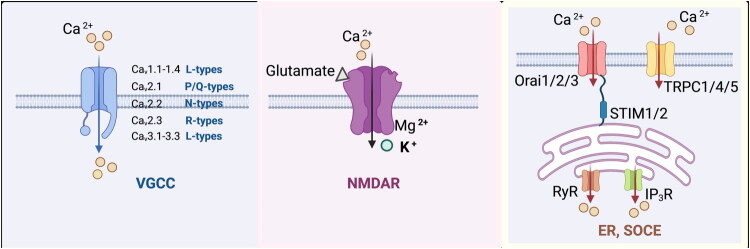
Calcium signalling activation. A) VGCCs mediate depolarization-induced calcium influx to regulate secretion, gene expression and neurotransmission. Cav1.1-1.4, L-type voltage-gated calcium-channel α1 subunits; Cav2.1, P/Q-type voltage-gated calcium-channel α1A subunit; Cav2.2, N-type voltage-gated calcium-channel α1B subunit; Cav2.3, R-type voltage-gated calcium-channel α1E subunit; VGCC, voltage-gated calcium channel family; B) NMDA receptors open upon depolarization and glutamate binding, permitting calcium and sodium entry. NMDAR, N-methyl-D-aspartate receptor. C) IP3 binds ER IP3 receptors to release calcium; store depletion activates STIM Orai SOCE. Orai1/2/3, pore-forming subunits of the store-operated calcium-release activated calcium (CRAC) channel; TRPC1/4/5, canonical transient-receptor-potential channels, voltage-independent, store-operated or receptor-operated calcium-permeable cation channels; RyR, ryanodine receptor; IP_3_R, inositol 1,4,5-trisphosphate receptor; ER, endoplasmic reticulum; SOCE, store-operated calcium entry; STIM1/2, stromal interaction molecules 1 and 2.The image was created using Biorender.

Calcium release from intracellular stores, triggered through activation of IP_3_Rs or RyRs on the ER membrane, leads to a decrease in ER calcium concentration. This depletion is sensed by stromal interaction molecules 1 and 2 (STIM1/2), which undergo conformational changes, oligomerize, and translocate toward the plasma membrane. There, STIM proteins physically interact with and open Orai1/2/3 calcium release–activated calcium channels, permitting a highly calcium-selective influx that replenishes ER calcium stores [16]. SOCE sustains intracellular calcium signalling and regulates gene expression through activation of CaM-dependent protein kinases and modulation of nuclear factor of activated T cells (NFAT) transcription factors. Additionally, SOCE promotes the recruitment of transient receptor potential canonical (TRPC1/4/5) channels to the plasma membrane, forming STIM1–TRPC complexes activated by the GPCR–PLC pathway. This process enhances nonselective cation influx, induces membrane depolarization, activates VGCCs, and synergistically amplifies calcium signalling [17–19]. The activation mechanisms of calcium signaling pathways are illustrated in Figure 1.

## 3. Dysregulation of calcium signalling pathways in depression

Extensive evidence from clinical studies and animal models demonstrates that calcium signalling pathways are disrupted in depression (Table 1).

**Table 1.** Dysregulation of calcium signalling pathways in depression.

Research stage	Patient/Model	Abnormal indicator	Brain region	Mechanism	Reference
**Preclinical Research Characteristics**	CSDS	L-VGCC up-regulating	HPC CA1	Potentiating the Ahnak scaffold	[24]
	CUMS	VGCC subunit α2δ-1 increasing	HT	VGCC enhancing NMDAR-gated channels	[25]
	C57BL/6J acute slice	Postsynaptic Ca²⁺ influx	LHB	NMDARs and subsequent CaMKII–PP1/PKA–GluA1 signalling	[26]
	CUS	Ca²⁺ influx	VHPC	Enhanced recruitment of IP3-sensitive Ca²⁺ stores	[28]
	CUS	Ca²⁺ influx	HPC	Enhanced NMDA-mediated Ca²⁺ influx; Calbindin-D28k decrease	[31]
**Clinical Research Characteristics**	MDD patient	*CACNA1C* rs1006737	HPC	Genetic vulnerability to depressive symptoms	[32,34–36]
	MDD patient	Calcium signal amplification	HPC and PFC	Abnormal functional connectivity between the hippocampus and prefrontal cortex	[37,38]
	MDD patient	Platelet free-calcium transient amplitude decreased	Venous blood	VOCC-IP₃R-SOCE is downregulated	[39]

CSDS, Chronic Social Defeat Stress; CUMS, Chronic Unpredictable Mild Stress; CUS, Chronic Unpredictable Stress; HPC,hippocampal; VHPC, ventral hippocampus; HT, hypothalamus; LHB, lateral habenula; PFC,prefrontal cortex; CACNA1C rs1006737；a single-nucleotide polymorphism (SNP) in the CACNA1C gene that encodes the Cav1.2 α1C subunit of L-type voltage-gated calcium channels).

### 3.1. Preclinical research characteristics

Aberrant calcium signalling is closely associated with the development of depressive-like behaviours in mice. Multiple calcium channels, including VGCCs, NMDA receptor–gated channels, and ER calcium-release channels, are dysregulated across various depressive mouse models. Genetic polymorphisms in L-VGCCs have been identified as important susceptibility factors for depression. Ahnak is an extremely large protein with a molecular weight of 680 kDa and was known as a binding partner of the β subunit of cardiac VGCC [20] and the regulation of L-type VGCCs by Ahnak has been reported in cardiomyocytes [21], osteoblasts [22], and T cells [23], implicating Ahnak in the regulation of neuronal L-type VGCC. In brain tissue from depressed mice, Ahnak interacts with the α1 and β subunits of L-VGCCs and with the p11/Anxa2 complex, forming a scaffold that links the p11/Anxa2 complex to L-VGCCs. Mice with whole-brain Ahnak deletion or glutamatergic neuron–specific Ahnak knockout in the forebrain display pronounced depressive-like behaviours, including reduced sucrose preference, anhedonia, increased immobility in forced swimming and tail suspension tests, and markedly reduced L-type calcium currents [24]. Calcium signalling pathways also interact with one another. Dysregulated VGCC expression can affect NMDAR-mediated calcium influx. For example, in a chronic unpredictable mild stress (CUMS) mouse model, expression of the VGCC auxiliary subunit α2δ-1 is increased in the hypothalamus. This upregulation enhances NMDAR1 expression and activity, resulting in reduced sucrose preference, prolonged immobility in behavioural despair tests, and reduced locomotor activity in the open-field test, all hallmarks of depressive-like behaviour [25]. Moreover, abnormally elevated neuronal activity in the lateral habenula (LHb) is strongly linked to depressive phenotypes. In this region, NMDARs and calcium-permeable AMPARs (CP-AMPARs) serve as major sources of calcium entry. Previous studies have shown that low-frequency stimulation induces NMDAR-dependent long-term depression (LTD) in both synaptic and extrasynaptic LHb regions. CP-AMPARs further potentiate NMDAR activation, thereby promoting the emergence of depressive-like behaviour [26].

The ER is essential for maintaining neuronal calcium homeostasis. Opening of VGCCs increases presynaptic calcium levels, which are subsequently amplified through ER calcium release *via* CICR, thereby regulating synaptic vesicle exocytosis. ER-derived calcium also modulates activity-dependent gene expression through activation of cAMP response element–binding protein (CREB) signalling pathways. Both of these physiological processes are impaired in depression [27]. ER calcium release is primarily controlled by IP_3_Rs and RyRs. In depressed mice, IP_3_R hyperactivation triggers excessive ER calcium efflux, disrupting synaptic plasticity and contributing to abnormal neuronal excitability [28]. Following ER calcium depletion, SOCE is activated to replenish ER stores by facilitating extracellular calcium influx. In depressive mouse models, enhanced SOCE activity leads to excessive calcium entry, promoting hippocampal neuroinflammation [29].

In addition to the direct dysregulation of calcium channels, several key downstream effector molecules associated with these channels exhibit marked abnormalities in depression. In a chronic restraint stress (CRS) mouse model, hippocampal brain-derived neurotrophic factor (BDNF) expression was significantly downregulated [30]. This reduction was accompanied by sustained potentiation of NMDA-NR2B receptor activity, producing a chronic Ca^2+^ overload that over-activates the Ca^2+^-dependent phosphatase calcineurin and suppresses BDNF transcription; levels of Ca^2+^-binding proteins (calbindin-D28k) were also decreased, further impairing Ca^2+^ homeostasis. Conversely, the NMDAR antagonist memantine or selective NR2B blocker Ro-25-6981 normalized Ca^2+^ uptake, restored BDNF expression and reversed stress-induced depressive-like behaviour. These findings indicate that NR2B-driven, protracted elevations in intracellular Ca^2+^ signalling underlie the depressive phenotype observed in chronically stressed rats [31].

### 3.2. Clinical research characteristics

Variants in calcium-signalling-related genes are linked to depression risk: the calcium voltage-gated channel subunit alpha1 C (*CACNA1C*), pivotal for activity-dependent Ca^2+^ influx, carries the SNPs rs11832738 and rs1006737 as risk alleles for major depressive disorder [32–34]. A large UK sample analysis showed that the rs1006737 allele in L-VGCCs significantly increases susceptibility to both schizophrenia (*p* = 0.034) and recurrent depression (*p* = 0.013) [35,36].

The hippocampus (HPC) and prefrontal cortex (PFC) contain numerous calcium signaling pathways that play central roles in emotional regulation. Functional magnetic resonance imaging studies have demonstrated abnormal functional connectivity between these regions in patients with depression [37]. Adolescents with first-episode MDD show reduced functional connectivity between the hippocampus and multiple prefrontal regions, providing indirect evidence of intracerebral calcium signalling disruption in depression. These findings suggest that amplification of calcium signals and their dysregulated control may weaken synaptic connections, leading to impaired cognitive and emotional functions[37,38]. Platelets have emerged as a practical ‘peripheral neuronal surrogate’ because they express many of the same calcium-handling proteins (STIM1, Orai1, IP_3_R, SERCA, PMCA) that neurons use to translate receptor activation into intracellular Ca^2+^ signals. After stimulation by serotonin, dopamine, glutamate or thrombin, the resulting platelet free-calcium transient amplitude is decreased in major depressive disorder, paralleling the blunted Ca^2+^/cAMP responses seen in central synapses [39].

A systematic review of calcium signalling dysregulation in patients with depression and in animal models has revealed widespread imbalances across behavioural, symptomatic, circuit, cellular, and molecular levels. The specific pathological mechanisms underlying these alterations are discussed in detail in the following section.

## 4. Pathophysiological mechanisms of depression mediated by calcium signalling dysregulation

Dysregulated calcium signalling contributes to the pathogenesis of depression through multiple interconnected mechanisms, including impaired synaptic plasticity and dendritic spine loss, excitation–inhibition imbalance and neuronal network dysfunction, reduced hippocampal neurogenesis, neuroinflammation, HPA axis dysregulation, and abnormal monoamine receptor expression. Protein expression changes related to calcium signalling pathways are summarized in Table 2.

**Table 2.** protein expression changes related to calcium signalling pathways in depression.

Pathway	Protein Name(s)	Regulation Direction	Brain Region/Cell Type	Patient/Specific Model	Reference
**Synaptic Plasticity Impairment and Dendritic Spine Loss**	SV2A	Down	aHPC/dlPFC/ACC	Patients with MDD	[47]
	L-VGCCs/NMDAR-2B	Up	HPC	CRS rat	[117]
	SERCA2/IP_3_R2/MCU/BDNF CaMKII/CREB	Down	PFC	Chronic stress rat	[30]
**Neuronal Excitation–Inhibition Imbalance**	Cav3.1	Up	Medial PFC	Chronic stress mice Cav3.1^−/−^ mice	[52]
	PGC-1α/TFAM/MCU/GLT-1	Down	Nucleus/Mitochondria/Plasmalemma	Astrocytes derived from iPSCs	[53]
	Synaptophysin/PSD-95	Down	Synapse/Plasmalemma	Neurons derived from iPSCs	
**Impaired Hippocampal Neurogenesis**	BDNF/TrkB	Down	HPC	Alcohol-induced depression rat	[56]
	CaMKII/p-CaMKII/PI3K/Akt/ERK1/2	Down	HPC	CUMS rat	[57]
**Triggering and Amplification of Neuroinflammation**	IP3R3-GRP75-VDAC1/NOX2/NLRP3	Up	Whole-brain microglia	CSDS mice	[60]
	CaN/NFATc1/NLRP3	Up	HPC microglia	CSDS mice	[63]
	PSD-95	Down			
	Orai1/ NF-κB/ IL-1/ IFN-γ/ IL-1α/ IL-6/ TNF-α/C3	Up	Cerebral cortex/HPC astrocytes	LPS-treated mice (intraperitoneal injection)	[65]
**Dysregulation of the HPA Axis**	Cav1.2/Cav1.3	Up	HPC CA1	Chronic corticosterone	[69,70]
	CRH	Up	PVN neurons (hypothalamus)	CSDS/CUMS mice	[71]
	GR/BDNF	Down			
	AMPAR/NMDAR	Up	PVN (hypothalamus)	CUMS rat	[73]
	PMCA/SERCA/GSH/SOD/ CAT/BDNF	Down	HPC	CUMS rat	[74]
	NOX2	Up			
**Dysregulation of Monoamine Neurotransmission**	5-HT/BrdU/DCX/CaMKII	Up	Serum and Hippocampus	CUMS rat	[84]
	BDNF/p-TrkB/p-CREB/P-CaMKII/PSD-95/syn1/p-mTOR/5-HT2A	Down	PFC/BLA/HPC/Hypothalamus	CCI/CIN models	[86]
	p-ERK, IL-1β, TNF-α	Up	Serum		
	5-HT_2_B	Down	HPC astrocytes	CUMS mice	[87]
	GP/GS	Up			

SV2A, Synaptic Vesicle Glycoprotein 2 A; aHPC, anterior Hippocampus; dlPFC, dorsolateral Prefrontal Cortex; ACC, Anterior Cingulate Cortex; MDD, Major Depressive Disorder; L-VGCC, L-type Voltage-Gated Calcium Channel; NMDAR-2B, N-Methyl-D-Aspartate Receptor subtype 2B; SERCA2, Sarco/Endoplasmic Reticulum Ca²⁺-ATPase 2; IP₃R2, Inositol 1,4,5-trisphosphate Receptor type 2; MCU, Mitochondrial Calcium Uniporter; BDNF, Brain-Derived Neurotrophic Factor; CaMKII, Calcium/Calmodulin-dependent Protein Kinase II; CREB, cAMP Response Element-Binding protein; Cav3.1, Calcium channel, voltage-dependent, T type, alpha 1I subunit; PGC-1α, Peroxisome Proliferator-Activated Receptor-γ Coactivator-1α; TFAM, Mitochondrial Transcription Factor A GLT-1, Glutamate Transporter 1; iPSCs, induced Pluripotent Stem Cells; PSD-95, Postsynaptic Density protein 95; TrkB, Tropomyosin Receptor Kinase B; PI3K, Phosphatidylinositol-3-Kinase; Akt, Protein Kinase B (Akt); ERK1/2, Extracellular signal-Regulated Kinases 1 and 2; IP₃R3, Inositol 1,4,5-trisphosphate Receptor type 3; GRP75, Glucose-Regulated Protein 75; VDAC1, Voltage-Dependent Anion Channel 1; NOX2, NADPH Oxidase 2; NLRP3, NOD-Like Receptor family Pyrin domain containing 3; CaN, Calcineurin; FATc1, Nuclear Factor of Activated T-cells, cytoplasmic 1; Orai1, Calcium Release-Activated Calcium Channel Protein 1; NF-κB, Nuclear Factor kappa-light-chain-enhancer of Activated B cells; IL-1, Interleukin-1;IFN-γ, Interferon-gamma; IL-1α, Interleukin-1 alpha; IL-6., Interleukin-6; TNF-α, Tumor Necrosis Factor-alpha; C3, Complement Component 3; Cav1.2, Calcium channel, voltage-dependent, L type, alpha 1 C subunit; Cav1.3, Calcium channel, voltage-dependent, L type, alpha 1D subunit; HPA, Hypothalamic-Pituitary-Adrenal (axis); CRH, Corticotropin-Releasing Hormone; PVN, Paraventricular Nucleus; CUMS, Chronic Unpredictable Mild Stress; GR, Glucocorticoid Receptor; AMPAR,α-Amino-3-hydroxy-5-methyl-4-isoxazolepropionic Acid Receptor; PMCA, Plasma Membrane Ca²⁺-ATPase; SERCA, Sarco/Endoplasmic Reticulum Ca²⁺-ATPase; GSH, Glutathione; SOD, Superoxide Dismutase; CAT, Catalase; 5-HT, 5-Hydroxytryptamine (Serotonin); BrdU, 5-Bromo-2’-deoxyuridine; DCX, Doublecortin; 5-HT₂B, 5-Hydroxytryptamine Receptor 2B; GP, Glycogen Phosphorylase; GS, Glycogen Synthase.).

### 4.1. Synaptic plasticity impairment and loss of dendritic spines

Alterations in synaptic plasticity, including long-term potentiation (LTP) and long-term depression (LTD), are decisively regulated by postsynaptic calcium transients [40]. During LTP, high levels of NMDAR-mediated calcium influx activate CaMKs, particularly CaMKII, which enhances synaptic strength by phosphorylating synaptic proteins, increasing BDNF expression, and promoting dendritic spine growth [30,41]. Conversely, LTD is induced by low levels of NMDAR-mediated calcium influx, which activates calcineurin (CaN), leading to reduced synaptic strength, spine shrinkage, and elimination [42,43].

**Figure 2.**
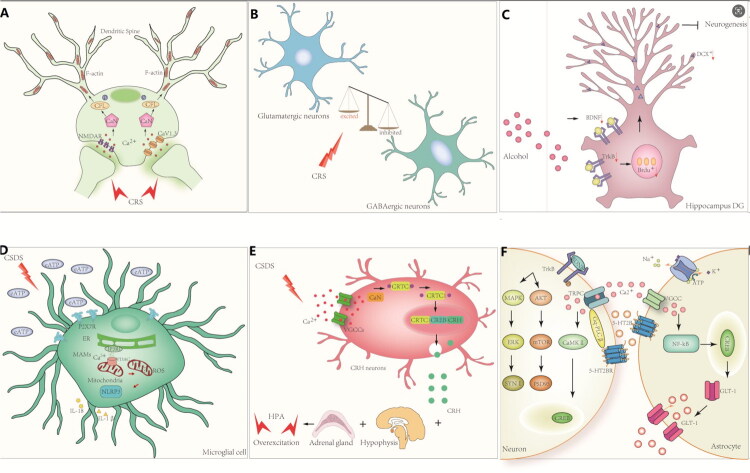
Calcium dyshomeostasis in depression. (A) Persistent cytosolic calcium elevation activates CaN and causes spine loss. CFL, Cofilin (actin-binding protein), CRS: Chronic Restraint Stress; CSDS, Chronic Social Defeat Stress; eATP, Extracellular Adenosine Triphosphate. (B) CRS increases calcium influx in medial PFC GABA neurons and disrupts excitatory inhibitory balance. CRTC1, CREB-regulated transcription coactivator 1; CREB, cAMP response element-binding protein. CRH, Corticotropin-releasing hormone. (C) BDNF TrkB suppression impairs hippocampal neurogenesis. CaN: Calcineurin; NF-κB: Nuclear factor kappa-light-chain-enhancer of activated B cells. (D) Microglial MAM activation boosts mitochondrial calcium, increasing ROS and NLRP3 inflammasome activity. MAMs: Mitochondria-associated membranes; NLRP3: NOD-like receptor family pyrin domain containing 3. (E) PVN calcium signalling stimulates CRH release and HPA axis overactivation. HPA: Hypothalamic-pituitary-adrenal (axis); GLT-1: Glutamate transporter 1. F) Neuronal 5 HT2B activation promotes CaMKII CREB BDNF transcription; astrocytic 5 HT2B enhances Kir4.1 buffering and GLT 1 mediated glutamate clearance. TrkB, tropomyosin-receptor kinase B； MAPK, mitogen-activated protein kinase；AKT, protein kinase B；VGCC, voltage-gated calcium channel；5-HT2BR, 5-hydroxytryptamine receptor 2B；mTOR, mechanistic target of rapamycin. CaMKI, calcium/calmodulin-dependent protein kinase I；NF-κB, nuclear factor kappa-light-chain-enhancer of activated B cells；CREB, cAMP response element-binding protein. SYNI, synapsin I；PSD95, postsynaptic density protein 95; GLT-1, glutamate transporter 1.The picture was drawn using the Adobe Illustrator 2023 version.

In chronic stress models, upregulated L-VGCCs and NMDAR-2B increase calcium influx, while downregulated SERCA2, IP3R2, and MCU impair calcium reuptake and buffering. This results in sustained high cytosolic calcium, activating CaN and mitochondrial apoptotic pathways. CaN dephosphorylates cofilin, severing F-actin and causing spine atrophy and loss. These structural changes are accompanied by reduced postsynaptic density, inhibition of CaMKII and CREB phosphorylation, and decreased BDNF expression, collectively impairing LTP and promoting LTD, leading to mood disorders and cognitive deficits [30,44,45] (Figure 2(A)).

Dysregulated calcium signalling triggers depression by dismantling dendritic spines and crippling excitatory synapses and plasticity. Dendritic spines play essential roles in processing, storing, and integrating synaptic inputs in projection neurons and serve as the principal sites for excitatory signalling in the brain. Activation of glutamate receptors within dendritic spines elicits substantial voltage changes and localized calcium transients in dendritic heads [46]. Human imaging/post-mortem data converge on hippocampal/cortical spine loss, reduced gray matter and impaired calcium integration [42,47]. In serotonin transporter (SERT) knockout mice exposed to chronic behavioural despair, increased calcium influx through activated postsynaptic calcium channels induces LTD, further weakening synaptic connectivity [40]. Similarly, certain antiepileptic medications with depressive side effects disrupt NMDAR-mediated calcium influx, enhance synaptic inhibition, and reduce synaptic transmission efficiency, thereby contributing to depressive symptoms [48].

### 4.2. Neuronal excitation–inhibition imbalance

Neuronal excitation–inhibition imbalance is a critical pathophysiological mechanism in depression, with calcium signalling functioning as a key regulator of neuronal excitability. Under normal conditions, neurons maintain a tightly controlled balance between excitatory and inhibitory inputs. In prefrontal cortical pyramidal neurons, the calcium-activated nonselective cation channel transient receptor potential melastatin 4 (TRPM4) is highly enriched in proximal dendrites, where it opens in response to increases in intracellular calcium. Calcium influx through TRPM4 channels establishes a ‘calcium–TRPM4–neuronal excitability’ positive feedback loop that amplifies depolarization and sustains neuronal firing [49]. Genetic knockout of *Trpm4* in mice further demonstrates the importance of calcium signalling in regulating neuronal excitability by selectively abolishing NMDAR-dependent LTP[50]. Excitation–inhibition balance is also regulated at the subcellular level within neurons. During low-intensity NMDAR activation, IP_3_-sensitive ER calcium stores in hippocampal cornu ammonis (CA1) dendritic spines amplify intracellular calcium signals through the mGluR–PLC–IP_3_ pathway, thereby reducing the threshold for LTD induction. Conversely, high-intensity stimulation leads to calcium-dependent inhibition of IP_3_Rs, preventing excessive ER calcium release. This mechanism helps limit unchecked synaptic strengthening and maintains overall synaptic plasticity [51].

In depression induced by chronic stress, the Cav3.1 T-type calcium channel becomes hyperfunctional in γ-aminobutyric acid (GABA) neurons of the medial PFC. This hyperactivity increases calcium influx and promotes burst firing in GABAergic neurons, thereby enhancing inhibitory tone within the PFC. The resulting excitation–inhibition imbalance contributes directly to the development of depressive-like behaviours. Conversely, Cav3.1 knockout (Cav3.1^−/−^) mice show resistance to stress-induced depressive phenotypes, and administration of a selective T-type calcium channel antagonist similarly reduces these behaviours [52]. Beyond neurons, dysregulation of astrocytic calcium homoeostasis also contributes to depression. Patients with depression exhibit mitochondrial dysfunction both centrally and peripherally, accompanied by disrupted calcium handling in astrocytes. Sustained elevations in cytosolic calcium, coupled with reduced mitochondrial calcium uptake, impair astrocytic glutamate clearance, leading to excessive glutamate accumulation in the synaptic cleft. This excess glutamate overactivates postsynaptic ionotropic glutamate receptors, causing neuronal excitotoxicity. Serotonin (5-HT) interventions have been shown to alleviate depressive phenotypes by restoring astrocytic calcium balance and improving mitochondrial function[53] (Figure 2(B)).

### 4.3. Impaired hippocampal neurogenesis

The HPC is a key brain region involved in cognitive function and emotional regulation and serves as a primary site of adult neurogenesis [54]. L-VGCCs regulate calcium influx in hippocampal neurons, thereby influencing neurite outgrowth, neuronal migration, and differentiation of neural stem/progenitor cells. These channels also contribute to the induction of LTP, BDNF release, and CREB-mediated transcriptional pathways [55]. The BDNF–tropomyosin receptor kinase B (TrkB) signalling pathway plays a central role in hippocampal neurogenesis, and TrkB activation is tightly regulated by calcium signalling [41]. In an animal model of alcohol-induced depression, chronic heavy drinking impaired the survival and differentiation of hippocampal neural precursor cells and diminished BDNF-mediated neurogenic effects. Treatment with TrkB agonists enhanced BDNF signalling, restored neurogenesis, and alleviated depressive-like behaviors. Although the study did not directly examine calcium signalling as a mediator of TrkB activation, calcium remains a critical upstream regulatory mechanism and is strongly implicated in depression associated with impaired hippocampal neurogenesis [56].

CaMKII, a key downstream effector of the calcium signalling pathway, also plays an essential role in hippocampal neurogenesis. Studies have shown that CaMKII expression is reduced in the HPC of individuals with depression, and both its phosphorylation and enzymatic activity are significantly inhibited. This suppression disrupts the survival, proliferation, and differentiation of neural stem cells by impairing major neurogenic signalling pathways, including PI3K/Akt and ERK1/2 [57] (Figure 2(C)).

### 4.4. Triggering and amplification of neuroinflammation

Neuroinflammation is a major pathogenic mechanism underlying MDD. Microglial activation is a critical event in this inflammatory cascade, and Ca^2+^ signalling serves as a central regulator of this process. Calcium overload impairs mitochondrial function and promotes the release of damage-associated molecular patterns (DAMPs) from stressed or injured neurons and glial cells. These DAMPs activate microglia, amplify neuroinflammation, and stimulate the secretion of proinflammatory mediators, such as high-mobility group box 1 and S100 calcium-binding protein β. Under Ca^2+^-dependent modulation, these mediators further recruit and activate immune cells, thereby sustaining and propagating the inflammatory response [58,59]. Mitochondria-associated ER membranes (MAMs) in microglia have been shown to be activated in both chronic social defeat stress (CSDS) mouse models and *in vitro* extracellular ATP stimulation experiments. This activation promotes Ca^2+^ transfer from the ER to mitochondria *via* the IP_3_R3–glucose-regulated protein 75 (GRP75)–VDAC1 complex. Excessive mitochondrial Ca^2+^ uptake reduces mitochondrial membrane potential, increases reactive oxygen species (ROS) production, and impairs mitochondrial function, ultimately leading to activation of the NOD-like receptor thermal protein domain–associated protein 3 (NLRP3) inflammasome. This cascade drives neuroinflammation and induces depression-like behaviours in mice [60]. Intervention studies further support this mechanism. Conditional knockout of GRP75 or pharmacological inhibition of the purinergic 2 × 7 receptor reduces ER–mitochondrial Ca^2+^ transfer and subsequent neuroinflammation, thereby alleviating depression-like behaviours [60–62]. Similarly, in CUMS models, microglia display elevated cytosolic and mitochondrial Ca^2+^ levels and increased CaN activity. This promotes the nuclear translocation of NFAT cytoplasmic 1 (NFATc1), driving microglial polarization toward the proinflammatory M1 phenotype. Concurrent NLRP3 inflammasome activation and ROS overproduction lead to the downregulation of synaptic proteins in hippocampal neurons and the emergence of depressive-like behaviours[63].

Calcium ions regulate microglial polarization, promote central neuroinflammation, and activate astrocytes to further contribute to the inflammatory response [64]. In a model of peripheral inflammation induced by intraperitoneal lipopolysaccharide (LPS) injection, astrocytes exhibited increased expression of the SOCE channel subunit Orai1. This upregulation led to calcium channel hyperactivation and elevated cytosolic and mitochondrial calcium levels, which in turn activated the NFATc1–nuclear factor kappa B (NF-κB) pathway. These changes were accompanied by significant enrichment of proinflammatory pathways, including interleukin-1 (IL-1), NF-κB, and interferon-γ (IFN-γ), and increased secretion of IL-1α, IL-6, and tumour necrosis factor-α (TNF-α). Moreover, astrocyte-derived complement component C3 activated neighbouring microglia, amplifying neuroinflammation and contributing to the development of depression-like behaviours [65] (Figure 2(D)).

### 4.5. Dysregulation of the HPA axis

The activity of the HPA axis is closely associated with emotional regulation. Hyperactivation of the HPA axis and impaired cortisol negative feedback mechanisms disrupt neuroplasticity, promote neuroinflammation, and negatively impact mood and cognitive function. Cortisol in humans and corticosterone in rodents are strongly implicated in the pathophysiology of depression [66]. These hormones exert their effects primarily through mineralocorticoid receptors (MRs) and glucocorticoid receptors (GRs). MRs help stabilize neuronal activity and mediate rapid stress responses, whereas GRs (activated predominantly under conditions of elevated hormone levels) facilitate stress adaptation and recovery [67,68].

The HPA axis influences neuronal function partly by modulating calcium channel activity. Under chronic stress, enhanced HPA axis activity increases neuronal excitability and susceptibility to depressive disorders by altering calcium signalling. Following stress exposure, elevated corticosteroid levels cross the blood–brain barrier (BBB) and bind to receptors that regulate gene transcription in hippocampal and amygdala neurons. VGCCs are among the primary downstream targets of corticosteroid signalling. Under basal corticosterone conditions, L-VGCC currents in hippocampal CA1 neurons remain relatively low. However, stress-induced activation of GRs leads to the formation of receptor homodimers that bind DNA and progressively increase L-VGCC current amplitude. The resulting increase in calcium influx following stress contributes to the transient normalization of neuronal activity. Nevertheless, persistent or excessive calcium entry poses significant risks: chronic elevation of intracellular calcium can induce neuronal excitotoxicity and structural damage, particularly in vulnerable regions such as the HPC and amygdala [69,70].

The salt-inducible kinase 1–CREB-regulated transcription coactivator 1 calcium signalling pathway within hypothalamic paraventricular nucleus (PVN) neurons has been shown to drive corticotropin-releasing hormone (CRH) hypersecretion, leading to the hyperactivation of the HPA axis and development of depressive-like behaviours. In CSDS or CUMS mouse models, sustained high-frequency firing of CRH neurons results in membrane depolarization and subsequent opening of VGCCs, causing a rapid increase in intracellular calcium levels. Elevated calcium levels activate CaN, which dephosphorylates CRTC1, allowing its translocation into the nucleus. Once inside the nucleus, CRTC1 forms a complex with CREB, binds to the CRH promoter, and promotes excessive CRH transcription and release. This cascade leads to hyperactivation of the HPA axis, followed by downregulation of GR and BDNF, impairment of synaptic plasticity, and emergence of depressive-like phenotypes [71].

In addition to the aforementioned pathways, studies using the chronic unpredictable stress protocol in rat models of depression have shown that CRH overexpression [72] leads to marked upregulation of GluR1 in the hypothalamic PVN [73]. This is accompanied by overactivation of NMDA receptors, sustained elevations in intracellular Ca^2+^ levels, and inactivation of Ca^2+^ pumps. The resulting cytosolic Ca^2+^ overload overwhelms mitochondrial buffering capacity, triggering excessive ROS production, depletion of glutathione (GSH), and inactivation of antioxidant enzymes such as superoxide dismutase (SOD) and catalase (CAT). This collapse of the antioxidant defense system contributes to hippocampal volume loss, decreased BDNF levels, and depression-like behaviour emergence in affected animals [74]. Moreover, excessive glutamatergic transmission further damages the neuronal cytoskeleton, exacerbating the development of depressive pathology [75–77] (Figure 2(E)).

### 4.6. Dysregulation of monoamine neurotransmission

Calcium signalling is a key downstream mediator of monoamine neurotransmitter systems, including 5-HT, DA, and norepinephrine (NE), and plays a central role in regulating neuronal signalling and excitability [78]. Calcium ions are essential for translating monoaminergic receptor activation into neuronal responses. Monoamine receptors and transporters modulate synaptic transmission, neuronal excitability, and synaptic plasticity in part by triggering calcium influx [79]. NE, for example, induces synchronized intracellular calcium elevations in astrocytes *via* activation of α1-adrenergic receptors on the astrocytic membrane [80]. In turn, astrocytes employ calcium-dependent mechanisms to regulate the release of neurotransmitters, including glutamate, thereby enhancing neuronal excitability and strengthening excitatory synaptic transmission [81,82].

Disrupted calcium signalling can induce hyperexcitability of 5-HT neurons, increase vulnerability to stress, impair monoaminergic neuronal function, and ultimately contribute to the development of depressive symptoms [83] In a CUMS-induced Sprague–Dawley rat model of depression, elevated serum and hippocampal 5-HT levels, increased bromodeoxyuridine/doublecortin (BrdU/DCX)-positive immature neuron counts, higher intracellular calcium levels, and increased CaMKII expression were observed [84]. Monoamine-targeting antidepressants further highlight the interplay between serotonin signalling and calcium pathways. In CUMS-induced C57 mice, SSRI administration produced robust antidepressant effects in the forced swim, sucrose preference, and novelty-suppressed feeding tests. Clinically, elevated serum BDNF levels were positively correlated with lower 17-item Hamilton Depression Rating Scale scores. A systematic mechanistic investigation revealed that SSRIs inhibit SERT upon entering the brain, rapidly increasing synaptic 5-HT levels. The resulting elevation activates 5-HT_2_B receptors on both neurons and astrocytes, initiating a cascade of calcium-dependent signalling events [53]. Although the specific long-term effects of SSRI treatment on calcium signalling in depression remain largely unexplored, studies in obsessive-compulsive disorder (OCD) have revealed that chronic SSRI administration elevates synaptic serotonin levels. This persistent stimulation triggers transcriptional changes, specifically upregulating the expression of Cav1.2 (L-type voltage-gated calcium channels) in astrocytes, effectively opening more valves for Ca^2+^ entry [85]. Future research should further investigate depression calcium signalling mechanisms to elucidate the precise molecular pathways linking long-term SSRI exposure to clinical recovery in depression.

In a study examining comorbid pain and depression [86], 5-HT_2_B receptor activation triggered the Gq–PLCβ pathway, resulting in the opening of Cav1.2/1.3 channels and SOCE components. This rapidly elevated intracellular calcium levels, which in turn activated CaMKII and promoted CREB nuclear translocation, thereby enhancing transcription of BDNF, activity-regulated cytoskeleton-associated protein (Arc), and synapsin. Subsequent BDNF binding to TrkB receptors activated the PI3K–Akt–mTOR and MAPK–ERK pathways, promoting postsynaptic density protein 95 (PSD-95) clustering, dendritic spine maturation, and synaptotagmin 1–mediated vesicle release. Collectively, these processes establish the molecular basis for LTP. Sustained BDNF–TrkB signaling also enhances hippocampal dentate gyrus neural stem cell proliferation and supports the survival of newborn neurons, thereby alleviating depressive behaviours. In astrocytes [87], 5-HT_2_B receptor activation induces a substantial calcium influx that rapidly increases Na^+^/K^+^-ATPase activity, restoring ion gradients and re-establishing Kir4.1-mediated potassium buffering. Subsequent activation of NF-κB and CREB upregulates glutamate transporter 1 (GLT-1), reducing extrasynaptic glutamate accumulation. Concurrently, astrocytes release ATP, glutamate, and D-serine to support synaptic plasticity and restore GABA_A_ receptor–mediated inhibition, thereby correcting the excitation–inhibition imbalance characteristic of depression (Figure 2(F)). Different types of glial cells exhibit distinct patterns of calcium signal fluctuations: astrocytes tend to be over-activated or silent, microglia manifest inflammation-driven ‘enhanced calcium oscillations’ whereas oligodendrocytes function more in the indirect regulation of myelin plasticity [60,88,89].

The preceding discussion highlights the central role of calcium signaling in regulating multiple pathophysiological processes involved in depression, including synaptic plasticity and dendritic spine integrity, neuronal excitation–inhibition balance, hippocampal neurogenesis, neuroinflammation, HPA axis dysregulation, and monoamine neurotransmission. The following sections examine how these calcium signalling–mediated mechanisms can be leveraged for diagnostic assessment and therapeutic intervention in depression.

## 5. Diagnostic and therapeutic potential of targeting calcium signalling

### 5.1. Calcium signalling as a biomarker for the diagnosis and prognosis of depression

#### 5.1.1. Specificity and sensitivity of peripheral calcium signalling in depression

Direct access to brain tissue in patients with depression is highly limited, and clinical symptom profiles frequently overlap with other neuropsychiatric disorders. Consequently, identifying reliable peripheral diagnostic biomarkers that can indirectly reflect central nervous system dysfunction is of considerable importance [90]. Platelets share several key signalling features with neurons. Although circulating in the periphery, platelets express neurotransmitter receptors (including 5-HT and α2-adrenergic receptors) that resemble those found on neurons. They also possess a complete phosphoinositide–calcium second messenger cascade (the PI–PLC–IP_3_–Ca^2+^ pathway). When platelets are exposed to neurotransmitters such as 5-HT, NE, or DA, receptor activation stimulates PLC, generating IP_3_ and triggering calcium release from ER stores, producing quantifiable intracellular calcium transients [39,90,91]. These calcium responses can be measured in real time using fluorescent calcium probes, allowing assessment of the amplitude, duration, and sensitivity of platelet calcium signalling [92].

Attenuated platelet calcium responses to 5-HT stimulation have emerged as stable, heritable, and diagnostically valuable peripheral trait markers for MDD. In a multicentre longitudinal clinical trial, venous blood samples were collected at baseline, 3 months, and 6 months. Using the fluorescent calcium indicator Fura-2 AM, researchers measured peak calcium transients (Δ[Ca^2+^]ᵢ) in platelets stimulated with 100 μM 5-HT. The Δ[Ca^2+^]_i_ in the MDD group was 34% lower than that in healthy controls, and this reduction remained significant throughout the 6-month follow-up period, even in patients who achieved clinical remission (Hamilton Depression Rating Scale score ≤ 7). These results indicate that diminished 5-HT–induced platelet calcium responses are present not only during acute episodes of MDD but persist after symptomatic remission, supporting their value as trait-like peripheral biomarkers [93]. However, these findings contrast with those of earlier studies. Prior investigations using the fluorescent dye Fura-2 to assess intracellular calcium responses in platelets reported that patients with MDD (evaluated during electroconvulsive therapy to minimize medication confounding) exhibited significantly enhanced calcium responses to 5-HT stimulation. Following successful antidepressant treatment, this initially enhanced platelet calcium response diminished and showed a positive correlation with clinical symptom improvement, indicating that it functions as a state marker of depression [94]. Additionally, PDZ and LIM domain–binding protein 5 (PDLIM5) is a small adaptor protein that regulates neuronal calcium conductance by interacting with protein kinase C-ε and the α-1β subunit of N-type calcium channels [95]. Earlier studies reported that PDLIM5 mRNA levels in leukocytes were significantly lower in patients with drug-naïve MDD compared with healthy controls. After 4 weeks of paroxetine treatment, PDLIM5 expression returned to normal and was associated with specific PDLIM5 SNPs. The rapid normalization of PDLIM5 expression indicates that it is a state-sensitive, calcium-related biomarker reflecting depressive status [96]. Recent evidence suggests that although peripheral calcium levels are associated with depression, their specificity is limited because the effects of inflammation and insulin resistance cannot be fully excluded. Several studies have reported reduced peripheral blood calcium levels in patients with MDD, with these decreases correlating with neurotoxicity markers and symptom severity. However, peripheral calcium alterations can also be influenced by systemic inflammation and insulin resistance, indicating that this phenomenon is not unique to depression [93].

The contradictory findings regarding platelet calcium signalling in depression may arise from earlier studies conceptualizing it either as a trait marker or a state marker. Future studies can adopt a double-blind, longitudinal design to record “resting” and “activated” states of calcium concentration, and comparing the differences between the two groups.

#### 5.1.2. Risk assessment based on calcium signalling pathway genes

At present, no definitive evidence supports the use of genetic risk scores based on calcium signalling pathway–related genes for diagnosing or predicting depression. Calcium channel blockers (CCBs), which target components of these pathways, are commonly prescribed for hypertension, a condition frequently comorbid with depression. Using Mendelian randomization, researchers examined whether genetically predicted CCB use influences depression risk. The analysis found no causal relationship between CCB use and depression [97]. Similarly, another study explored the phenotypic expression of genetic susceptibility to MDD using a polygenic risk score (PRS) derived from genome-wide association studies. Although the PRS for MDD showed associations with several phenotypes, it explained only a very small proportion of phenotypic variance. The findings highlight the need for much larger discovery and target samples to improve the predictive utility of PRS and to better understand how genetic risk for depression manifests [98].

#### 5.1.3. Application of emerging calcium-related technologies in in vivo and in vitro diagnosis

Advanced technologies such as calcium imaging, genetically encoded calcium sensors, and patch-clamp electrophysiology provide high temporal and spatial resolution for assessing central and peripheral calcium signalling relevant to depression [99,100]. For example, two-photon calcium imaging enables real-time visualization of neuronal calcium activity *in vivo*. Using this technique, researchers observed significantly reduced Ca^2+^ activity in pyramidal neurons of the temporal cortex during the depressive phase of BD, with activity levels closely correlating with depressive-like behaviours [101]. The integration of optogenetics and fibre photometry has further become a powerful approach for elucidating calcium dynamics within specific neural circuits and glial populations in animal models of depression. In chronically stressed mice, calcium indicators expressed in defined brain regions combined with optic fibre implantation allow real-time recording of calcium transients during optogenetic activation or inhibition. For instance, optogenetic activation of LHb astrocytes produced approximately a 30% ΔF/F increase in Ca^2+^ peak amplitude, positively correlating with depressive-like behaviours [102]. Likewise, optogenetic stimulation of LHb glutamatergic neurons induced a 45% ΔF/F increase in calcium signals and elevated firing frequency, both of which were reversible with optogenetic inhibition [103]. These findings not only demonstrate aberrantly enhanced calcium signalling in depression-related circuits but also provide causal evidence that manipulating calcium dynamics can reverse depressive phenotypes. Real-time electroencephalography (EEG) also holds promise for objective depression diagnosis. A recent innovation involves integrating an astrocyte-augmented spiking neural network into EEG-based diagnostic models. By incorporating astrocytic calcium wave dynamics to strengthen long-range signal representations, this approach significantly improves EEG pattern recognition and supports earlier and more objective identification of depression [104]

Furthermore, computerized neuropsychological assessments provide additional evidence supporting calcium signalling as a potential diagnostic marker. In patients with depression, serum calcium levels were significantly and positively correlated with composite cognitive scores, processing speed, executive functioning, and global functioning, supporting serum calcium as a potential peripheral biomarker for depression [125].

### 5.2. Drugs targeting calcium signalling in depression therapy

#### 5.2.1. CCBs

Current evidence regarding the relationship between CCBs and depression remains inconsistent. A large-scale study reported that the sustained use of CCBs was associated with a lower incidence of depression [105,106]. Similarly, a large prescription event monitoring study conducted in UK general practice found that the incidence of depression among patients taking diltiazem or nicardipine was comparable to that of patients using ACE inhibitors (diltiazem: 1.92/1000 patient-months; nicardipine: 1.62/1000 patient-months), with rate ratios of 1.07 (0.82–1.40) and 0.86 (0.69–1.08), respectively, indicating no increased risk of depression with CCB exposure [107]. Conversely, a meta-analysis indicated that CCBs may increase the risk of depression [108].

The inconsistent reports on calcium-channel blockers (CCBs) and depression stem from several intertwined factors: differing study designs (observational vs. randomized trials), variations in drug class and dosage (di-hydropyridines have low BBB penetration, whereas non-di-hydropyridines such as verapamil can affect neuronal calcium homeostasis), channel-subtype selectivity (L-type vs. T-type), and patient heterogeneity (comorbid cardiovascular disease and genetic differences in drug metabolism) [109]. Controlling these variables within a unified framework, or using stratified randomized trials that compare specific CCB subtypes, is essential to clarify the true impact of CCBs on depressive disorders.

#### 5.2.2. NMDAR antagonists

Ketamine, a well-characterized NMDAR antagonist, exhibits rapid antidepressant effects that have been robustly validated in preclinical models. Its mechanism of action is closely linked to the modulation of calcium signaling. Studies have shown that ketamine’s rapid antidepressant response is mediated by its ability to block NMDAR-dependent burst firing in the LHb. This pathological burst activity depends on both NMDARs and low-voltage–activated T-type calcium channels. Notably, local inhibition of either channel in the LHb is sufficient to produce rapid antidepressant effects in rodent models [110].

Furthermore, ketamine likely exerts its antidepressant effects through multiple mechanisms, including selective inhibition of NMDARs containing the NR2B subunit, suppression of eukaryotic elongation factor 2 kinase phosphorylation, enhancement of BDNF/TrkB signalling, and activation of the mTOR pathway. Many traditional Chinese medicines also act on the BDNF/TRKB pathway [^111^]. Additionally, one of its metabolites, R-hydroxynorketamine, has been shown to produce antidepressant-like effects independently of NMDAR blockade [112].

Furthermore, the U.S. Food and Drug Administration has approved esketamine (which possesses approximately fourfold higher affinity for NMDARs than racemic ketamine) as a nasal spray for the treatment of treatment-resistant depression. Preliminary clinical studies have shown that single or repeated intravenous doses of ketamine can produce rapid antidepressant and antisuicidal effects [113]. Although the clinical use of ketamine and its derivatives continues to expand, their long-term safety profiles and the durability of their therapeutic benefits require further thorough investigation.

#### 5.2.3. Lithium salts

Lithium is widely used for treating depression, particularly as an augmentation strategy when antidepressant monotherapy provides insufficient benefit. It is highly valued for its distinctive antisuicidal properties and its efficacy in preventing recurrent unipolar depressive episodes [114,115]. Lithium exerts its antidepressant effects by modulating intracellular calcium homeostasis through multiple mechanisms. These include inhibition of glycogen synthase kinase-3β, the phosphatidylinositol pathway, and protein kinase C, collectively reducing intracellular calcium release, decreasing neuronal excitability, and influencing synaptic plasticity. Additionally, lithium enhances calcium-related neuroprotective signalling by increasing the expression of BDNF and the antiapoptotic protein B-cell lymphoma 2, thereby promoting neuronal survival and synaptic function while limiting apoptosis [115]. A clinical case report further supports its therapeutic potential: the addition of lithium (900 mg/day, serum concentration 0.6 mmol/L) to existing antidepressant therapy produced marked clinical improvement within 4 weeks in a patient with unipolar depression. Full remission was achieved within 2 months and maintained for 1 year, allowing the patient to return to work, with no relapse observed after lithium discontinuation. Despite the need for regular serum monitoring, lithium remains a highly effective, rapid-acting, and cost-efficient augmentation option. It is particularly suitable for individuals with treatment-resistant depression, recurrent episodes, or a strong family history of mood disorders and warrants greater consideration in clinical decision-making [116].

### 5.3. Emerging therapeutic targets and strategies

With increasing clinical demand for improved depression treatments and a growing understanding of calcium signalling–related pathological mechanisms, several novel therapeutic targets and strategies have begun to emerge. These include the development of highly selective, brain-permeable Cav1.2 and Cav1.3 inhibitors or modulators; agents targeting RyRs or ER calcium stores to reduce ER stress and prevent calcium leakage; modulators of downstream calcium-dependent signalling effectors; approaches aimed at regulating calcium signalling microdomains; and development of inhibitors targeting SOCE.

#### 5.3.1. Precise targeting of specific VGCC subtypes

VGCCs are key regulators of neuronal excitability and neurotransmitter release, making their antagonism or modulation a promising strategy for developing novel antidepressants. Several existing antidepressants (e.g. SSRIs, SNRIs, and esketamine) exert secondary inhibitory effects on L-VGCCs, thereby reducing excessive calcium influx and neuronal hyperexcitability and alleviating depressive symptoms [4]. Among L-VGCCs, Cav1.2 and Cav1.3 are the predominant isoforms implicated in mood regulation. Studies have shown that Cav1.2 channel expression and L-type calcium currents are significantly elevated in chronic stress–induced rat models of depression [117]. Additionally, Cav1.3 channels in the ventral tegmental area activate CP-AMPAR mechanisms in the nucleus accumbens, contributing to cocaine-induced depression-like behaviours and highlighting Cav1.3 as a potential therapeutic target for depression and related psychiatric disorders [118]. These findings underscore the therapeutic potential of developing highly selective, brain-permeable Cav1.2/Cav1.3 inhibitors or modulators. Such compounds may offer more precise and effective antidepressant actions than current pharmacotherapies, warranting intensified efforts toward their screening, refinement, and clinical evaluation.

#### 5.3.2. Regulation of ER calcium homoeostasis

The regulation of ER calcium homeostasis has emerged as a promising therapeutic strategy for depression. In astrocytes, IP_3_ mediates ER calcium release primarily through IP_3_R2, the dominant isoform in these cells. Although genetic deletion of IP_3_R2 exerts minimal effects on neuronal calcium signalling, it profoundly disrupts astrocytic calcium dynamics. Accordingly, IP_3_ receptor antagonists may hold therapeutic potential for depression and chronic pain by inhibiting ER calcium release, reducing inflammatory mediator production, and attenuating neuroinflammation [28]. Contrary to receptor antagonism, certain natural compounds help stabilize ER calcium homeostasis. For example, the natural molecule saikosaponin B2 (SSB2) has been shown to regulate intracellular calcium levels. In LPS-induced microglial cells and a CUMS-induced depression model, SSB2 alleviated ER stress and maintained calcium balance by regulating intracellular Ca^2+^ levels. It simultaneously inhibited neuroinflammation and ferroptosis by reducing lipid peroxidation and intracellular iron accumulation, ultimately improving depressive-like behaviours [119]. Collectively, these findings suggest that both synthetic agents and natural compounds can modulate ER calcium homeostasis to influence mood regulation, offering novel therapeutic targets and strategic directions for future depression treatments.

#### 5.3.3. Targeting downstream effector molecules

CaMKs are widely expressed in neurons and glial cells throughout the brain and play essential roles in cortical functions, including cognition and memory. Dysregulation of these kinases (particularly CaMKII and CaMKIV) has been implicated in the pathogenesis of depression [120,121]. Reduced activity of CaMKII and CaMKIV is closely associated with cognitive impairment and depression-like behaviours in animal models. Recent evidence highlights the therapeutic potential of modulating these downstream effectors. Zhong et al. reported that Jujuboside A (JuA), an active compound extracted from Ziziphi Spinosae Semen, alleviated depressive-like behaviours and cognitive deficits in a CUMS rat model. JuA treatment increased monoamine neurotransmitter levels in both serum and HPC and reduced the number of immature neurons in the HPC. Mechanistically, JuA decreased intracellular calcium levels and CaMKII expression in immature neurons while enhancing synaptic density and dendritic complexity in the ventral dentate gyrus. Through these actions, JuA helps restore calcium homeostasis and synaptic plasticity, thereby exerting antidepressant effects [84].

#### 5.3.4. Modulation of calcium signalling microdomains

Targeting calcium signalling microdomains within specific organelles or synaptic sites represents an emerging direction in antidepressant development. Because the BBB limits the efficient delivery of conventional therapeutics, bioinspired nanodelivery systems, such as nanozymes, cell membrane–derived carriers, and exosomes, have gained attention for their ability to enhance targeting, improve bioavailability, and modulate complex intracellular signalling pathways by leveraging natural biological processes [122]. Ligand-modified nanocarriers further increase intracerebral drug utilization and offer targeted delivery capabilities [123]. For example, a borneol-modified polyethylene glycolated graphene oxide nanocarrier was shown to effectively penetrate the BBB by opening tight junctions and inhibiting efflux transporters. When loaded with ginsenoside Rg1, this delivery system significantly improved anhedonia, behavioural despair, and anxiety-like behaviours in a rat model of depression [126]. Researchers coupled mesoporous silica nanoparticles (NPs) with hyaluronic acid (HA), labelled the surface with a bifunctional peptide (Ang-2-Con-G, AC), and developed ketamine-loaded nanomaterials AC-RM@HAMS-KA NPs that can cross the blood-brain barrier, target N-methyl-D-aspartate (NMDA) receptors, and exert antidepressant effects [124]. Another study developed a hyaluronic acid nanogel–exosome composite system (HA NGs@exosomes) that, when administered intranasally, targets the brain, activates BDNF and exerts antidepressant effects [122]. Although nanomaterials can cross the blood–brain barrier, offer higher targeting efficiency and improved bioavailability, their clinical use as novel drug carriers is still limited, and further experimental evidence is required to establish their long-term safety.

#### 5.3.5. SOCE inhibitors

SOCE inhibitors represent a promising emerging class of antidepressant candidates. Mast cells have been implicated in the progression of depression through their contribution to neuroinflammation, and SOCE plays a critical regulatory role in this process [29]. Recent studies have demonstrated that the natural flavonoids quercetin and kaempferol exert antidepressant-like effects by suppressing TNF-α expression and secretion and inhibiting SOCE-mediated calcium influx. Notably, coadministration of either quercetin or kaempferol with the classical ORAI channel inhibitor YM58483 did not produce an additive inhibitory effect, suggesting that these flavonoids act through mechanisms similar to those of established SOCE inhibitors [29]. The content on the diagnostic and therapeutic potential of targeting calcium signalling is summarized in Table 3.

**Table 3.** Diagnostic and therapeutic potential of targeting calcium signalling.

Application	Target	Specific methods	Calcium response	Mechanism	References
**Biomarker**	Blood platelet calcium response	Venous blood	Down	Trait marker	[93]
		Venous blood	Up	State marker	[94]
	Diagnostic technology	Two-photon calcium imaging	/	Positive correlation	[101]
		Optogenetics and fiber photometry	/	Positive correlation	[102]
		Real-time electroencephalography	/	Astrocytic calcium waves establish long-range connections	[104]
		Computerized neuropsychological testing-serum calcium	/	Positive correlation	[125]
**Targeted drug**	Calcium channel blockers	Diltiazem Nicardipine	/	No correlation	[105,106]
	NMDAR Antagonists	Ketamine	Down	NMDA receptor-mediated calcium channels T-VSCCs	[110,113]
	Lithium Salts	Lithium Salts	Down	Inhibit GSK-3β, PI, PKC and increasing BDNF and Bcl-2 reducing intracellular calcium release	[115–117]
**Emerging strategies**	Precise Targeting of Specific VGCC Subtypes	Cav1.2/Cav1.3 inhibitors or modulators	Down	Cav1.2 and Cav1.3	[117,118]
	Regulation of ER Calcium Homeostasis	saikosaponin B2; IP3R2 antagonist	Down	IP3R2	[28,119]
	Targeting Downstream Effector Molecules	Jujuboside A	Down	CaMKI, CaMKII, and CaMKIV	[120,121]
	Modulation of Calcium Signaling Microdomains	GO-PEG-BO carried ginsenoside Rg1	Down	Nanozymes, cell-membrane systems, and exosomes	[122,123]
	SOCE Inhibitors	Flavonoids quercetin and kaempferol	Down	Inhibiting SOCE-mediated calcium influx	[29]

Ca²⁺, Calcium ion; a universal intracellular second messenger; NMDAR, N methyl D aspartate receptor; a calcium permeable ionotropic glutamate receptor; T VSCCs, T type voltage sensitive calcium channels; low voltage activated calcium channel subtype; GSK 3β, Glycogen synthase kinase 3 beta; a serine/threonine kinase linked to apoptosis and inflammation; PI, Phosphoinositide signalling pathway (general term); PKC, Protein kinase C; a calcium and phospholipid dependent kinase; BDNF, Brain derived neurotrophic factor; supports neuronal survival and plasticity；Bcl-2, B-cell lymphoma 2； Cav1.2 and Cav1.3, L-type voltage-gated calcium-channel α1C and α1D subtypes; VGCC, Voltage gated calcium channel (family); ER, Endoplasmic reticulum; IP₃R2-Type-2 inositol 1,4,5-trisphosphate receptor; CaMKI / CaMKII / CaMKIV, Calcium/calmodulin dependent protein kinases I, II and IV; GO-PEG-BO, Graphene oxide polyethylene glycol benzoxazine nanocarrier platform for drug delivery; ginsenoside Rg1, A neuroprotective saponin extracted from Panax ginseng; SOCE, Store operated calcium entry.).

## 6. Research prospects

Targeting calcium signalling has emerged as a promising therapeutic axis in depression research. Therapeutic focus has shifted from bulk neuronal populations to defined glutamatergic and GABA-ergic subtypes, with microglial and astrocytic calcium dysregulation now established as a core driver of depression pathogenesis. Supporting this, genome-wide data implicate calcium-channel and calcium-signalling gene variants in disease risk, and integrated analysis of these genetic signatures with functional phenotypes (e.g. peripheral calcium flux) can stratify patients for calcium-targeted precision therapy. To validate and refine these targets, high-resolution *in vivo* calcium-imaging tools that resolve circuit-specific dynamics at cellular or subcellular scale are urgently needed. Finally, to address blood–brain barrier permeability—the critical delivery bottleneck—nanocarriers, exosome-based miRNA delivery, and other targeted platforms that direct therapeutics to specific brain regions, cell types, or calcium microdomains represent the most viable strategies to enhance efficacy while limiting adverse effects.

## 7. Conclusion

Dysregulation of neuronal calcium signalling is a key factor in the pathogenesis of depression. Advances in calcium-signalling research may offer new directions and strategies for developing antidepressant therapies that specifically target these pathways; however, several challenges and limitations remain. First, although peripheral calcium responses (e.g. platelet calcium transients) are attractive potential biomarkers, their diagnostic specificity is confounded by variables such as inflammation and metabolic status, and further experimental validation is required. Second, while manipulating calcium-signalling pathways produces antidepressant-like effects in animal models, the reproducibility and safety of these findings in clinical settings—especially during chronic administration and across individuals—have yet to be established. Most importantly, calcium-targeted agents such as Cav1.2/Cav1.3 inhibitors are still in the exploratory phase of antidepressant development; their clinical utility lacks support from large-scale, randomized controlled trials. Rigorous translational studies are therefore essential to propel calcium-targeted interventions from bench to bedside.

## Chemical compounds studied in this article

**Ketamine,** PubChem CID: 3821; **Esketamine,** PubChem CID: 182137; **Lithium,** PubChem CID: 3028194; **Saikosaponin B2,** PubChem CID: 21637642; **Jujuboside A,** PubChem CID: 51346169; **Ginsenoside Rg1,** PubChem CID: 441923; **Quercetin,** PubChem CID: 5280459; **Kaempferol,** PubChem CID: 5280863; **YM58483,** PubChem CID: 2455.

## Data Availability

Data sharing is not applicable to this article because no datasets were generated or analyzed during the study.
